# Progress of island health in the Maldives

**DOI:** 10.1002/puh2.114

**Published:** 2023-08-12

**Authors:** Lawson Ifeanyi Eya, Imran Mohamed Adam, Muiz Ibrahim, Adriana Viola Miranda, Subbaram Kannan, Don Eliseo Lucero‐Prisno

**Affiliations:** ^1^ Noonu Atoll Hospital Manadhoo Maldives; ^2^ Global Health Focus Abuja Nigeria; ^3^ Gomel State Medical University Gomel Belarus; ^4^ International Higher School of Medicine International University of Kyrgyzstan Bishkek Kyrgyzstan; ^5^ Global Health Focus (GHF) Asia Bandung Indonesia; ^6^ Department of Microbiology School of Medicine The Maldives National University Male Maldives; ^7^ Department of Global Health and Development London School of Hygiene and Tropical Medicine London UK; ^8^ Faculty of Management and Development Studies University of the Philippines (Open University) Los Baños Laguna Philippines

**Keywords:** asia‐pacific, global health, health equity, island health, the Maldives, telemedicine

## Abstract

The provision of equitable healthcare on remote islands is critical in the fight to attain the sustainable development goals (SDGs). Many island countries, particularly the Small Island Developing States (SIDS), are confronted with numerous obstacles to accessing quality healthcare. Many SIDS are located in the Asia‐Pacific region, and they suffer shared challenges such as a lack of infrastructure, logistical concerns, and a shortage of health staff. As a SIDS, the Maldives has made significant progress in the health sector, when compared to its neighbours. Part of this is due to spending a large portion of the national budget on the social sector, and a well‐structured health system. Despite these accomplishments, it continues to struggle to provide equitable health care across the whole nation, especially to the small and underpopulated islands. One contributing factor is the dispersed geography. The pandemic highlighted the importance of telemedicine and its capacity to bridge the barriers to healthcare delivery, and its potential in the Maldives is huge. There is also a need to strengthen integrated health services through primary healthcare, train a larger workforce, and upgrade hospitals to be able to provide comprehensive medical services. This will limit the frequency of travel to the capital and abroad in search of better healthcare.

## INTRODUCTION

The concept of island health has become increasingly popular in recent decades. It focuses on strengthening the delivery of healthcare to islands, especially the remote and isolated ones. Many island nations face a plethora of public health challenges, especially those that make up the small island developing states (SIDS). These countries face other economic, social, and environmental issues, which are part of the rationale for classifying them as developing states by the United Nations (UN) [1, 2]. The UN has listed 58 SIDS, which are mostly found in these regions: the Caribbean; the Pacific, the Atlantic and the Indian Ocean [1]. The geography of these regions exposes them to frequent natural disasters and other ill effects of climate change, with some states even facing existential threats [1]. Several meetings and conferences have been organised by the UN in a bid to help these nations contain the worsening climate crisis and achieve sustainable development, most notably the SAMOA Pathway [1].

Recently, the first SIDS global health summit was held by the WHO, where the WHO member states convened to exclusively discuss issues of health and well‐being and collaborative efforts to improve on them [3]. Some of the shared challenges between SIDS are a lack of health infrastructure, difficulties with the logistics of medications and health equipment and, finally, a scarcity of health workers due to an inability to train and retain them [2]. These challenges present an obstacle in the way of achieving universal health coverage which is recognised as one of the components of the sustainable development goals (SDGs) [4].

The Asia‐Pacific region has over 100,000 small islands; though many are uninhabited. The health, communication and development problems of the inhabited islands make healthcare delivery challenging [5]. Some of the small islands in this region may be isolated from advanced health services, and with a small population, modern economic development seems unattainable [5]. This problem is reflected in the economies of these island nations [5]. The Asia‐Pacific Academic Consortium for Public Health held a symposium in Okinawa in 2009 that centred on how to provide healthcare to both metropolitan areas and rural and isolated islands in the Asia‐Pacific region [5].

## HEALTHCARE IN THE MALDIVES

The Maldives is a small island country located in the Asia‐Pacific region with over a thousand low‐lying islands dispersed in the Indian Ocean with a total population of 515,122 according to the last census in 2022 [6].

Before the introduction of modern healthcare in the Maldives, traditional medicine and healing, referred to as ‘Dhivehi beys’ or ‘Hakeemee beys’, were practised using both spiritual and herbal medicinal methods [7]. Since the establishment of the constitution in 1932, health has always been a top priority for the Maldives [8], and they have come a long way since the introduction of modern medicine. The Maldives has outperformed many of its neighbours in health outcomes, and it is the only South Asian country to achieve all its Millennium Development Goals and mortality‐based SDGs ahead of schedule [7]. It presently allocates more than 40% of the national budget to social sector spending [8], and it has the highest public spending on health in South Asia [7]. Healthcare for all Maldivians is free and funded by a universal health insurance scheme called ‘Aasandha’ [7, 9].

The Maldives has a well‐organised healthcare system that is arranged in a four‐tier hierarchical order with a health facility on every inhabited island [7, 9]. The health centres provide primary care; the atoll and regional hospitals are categorised as secondary care providers; and the Indira Gandhi Memorial Hospital serves as the country's leading public tertiary hospital [7, 9]. The public sector is the main health service provider and is supported by many private hospitals and non‐governmental bodies, which are concentrated in the greater Malé region [7, 9].

## CHALLENGES OF ISLAND HEALTH

The COVID‐19 pandemic exposed deficiencies in different health systems, including the Maldives. Health service delivery is still inequitable in the Maldives, despite government efforts [7]. Healthcare on many local islands, though easily accessible, is not as advanced as care in the greater Malé region [7, 9]. This is due to the concentrated population in this region, hence the development of better healthcare [9]. A majority of the population in the Maldives is based in the capital city, Malé (41%), whereas the remaining is dispersed across the administrative islands, resorts and industrial islands [6]. Many local islands lack the economic capacity to attract development, which in turn leads to migration away from these places in search of better jobs, education and sometimes access to better healthcare.

The dispersed geography of the country in the ocean makes logistics very challenging. Since the early days of modern medical care in the Maldives, Maldivians have faced difficulties travelling to other islands for medical care [8]. Although the situation has improved in recent times with the availability of the sea ambulance service, which provides emergency transport between islands, it has not completely disappeared. For instance, it can be extremely difficult to transport an unstable patient by sea ambulance during stormy weather. Some patients in the northern and southern regions prefer to travel abroad for treatment rather than spend the money on transport to Malé [7]. The Maldives has one of the highest spending per capita on medical travel among the SIDS [4]. Medical tourism is popular in the Maldives as it can be seen as prestigious and valuable, and people combine accessing medical care with other non‐health‐related agendas such as holidays. The Maldives is one of the highest importers of health services [4], but it sometimes suffers from the unavailability of health workers. Until recently, medical training was not offered in the Maldives, which meant that all medical doctors had to be trained abroad [10]. A significant percentage of health workers are foreign employees, and there is a high turnover of doctors, especially in the atoll clinics [10]. One reason is limited career and professional development [9] and the inability to cope in remote islands. Many local health workers are also not keen on working on some of these islands. This high turnover of doctors may also affect the building of a trust‐based patient–doctor relationship.

## OPPORTUNITIES TO IMPROVE

The pandemic also highlighted the importance and potential of telemedicine in strengthening healthcare delivery to people in distant locations. In the Maldives, telemedicine was effectively used to bridge barriers to accessing healthcare during the pandemic, and there is a high demand for expanding telemedicine to the atolls [7]. Telemedicine can be beneficial for non‐urgent medical care, medication refills, counselling and so on, and it will mostly benefit the elderly, people with disabilities and others who might face physical barriers to accessing healthcare. The Maldives needs to train a larger workforce to handle the increasing health burden and build a more resilient healthcare system. A larger workforce also ensures that vulnerable groups, including the elderly, children and migrant workers, are offered quality care. Health workers should be trained to develop strong capacity building for public health and on health issues rooted in climate change. The Maldives National University, which is located in the capital city Malé, is the sole provider of medical training for doctors in the country; therefore, more campuses could be created in different regions, and the government can also support other universities in the country to improve their standards and ability to deliver medical training.

It is important to prioritise integrated health service delivery, and this starts with strengthening primary healthcare. Health services in health centres have improved over the years. Routine outreach services are organised by the atoll and regional hospitals to health centres to enable patients to access specialist consultations, and some health centres in populous islands even have a few specialists; however, the least populous islands still struggle. The government could provide incentives for doctors to work on the remote islands. Even with universal healthcare, Maldivians still travel to Malé to access better healthcare; therefore, it is important to upgrade the regional hospitals to match the delivery of some of these services that are available in the capital. This may prove to be cost‐effective, and it may also curb the need for medical tourism.

## CONCLUSION

Many small island countries face a plethora of health challenges, and this situation is worse for some of the countries that make up the SIDS. Some of these challenges stem from their geography, remoteness and economic strength. The Asia‐Pacific region houses a lot of these SIDS, such as the Maldives. The Maldives has made great strides in the health sector as their health indicators have significantly improved over the years; however, some of these health challenges are still present. Despite having a well‐structured health system, healthcare delivery is still difficult, mainly due to the dispersed geography. In addition, they are also threatened by climate change. Telemedicine presents a great opportunity to bridge this gap, and there is a need for the government to invest in this area. They also need to strengthen the health system by increasing the workforce and upgrading the hospitals in different regions of the country to make quality healthcare more accessible to the remote islands. It is also important to keep in mind the threat of climate change in this country while making these changes.

## AUTHOR CONTRIBUTIONS

*Writing – original draft; writing – review and editing*; Lawson Ifeanyi Eya. *Writing – original draft*: Imran Mohamed Adam. *Writing – review and editing*: Muiz Ibrahim and Adriana Viola Miranda. *Supervision*: Subbaram Kannan. *Conceptualization; supervision*: Don Eliseo Lucero‐Prisno III.

## CONFLICT OF INTEREST STATEMENT

Miranda, Adriana is an Editorial Board member of Public Health Challenges and a co‐author of this article. Lucero‐Prisno III, Don‐Eliseo is the Editor‐in‐Chief of the journal and co‐author of this article. To minimise bias, they were excluded from all editorial decision‐making related to the acceptance of this article for publication.

## FUNDING INFORMATION

No funding was received for this manuscript.
